# Complete Genome Sequence of Finnry, a Subcluster L3 Mycobacteriophage from Charleston, South Carolina

**DOI:** 10.1128/mra.00636-22

**Published:** 2022-08-15

**Authors:** Christine A. Byrum, Véronique A. Delesalle, Claudia L. Gold, Daniel J. Bennett, B. Conner Fox, Brandon M. Houston, Harrison E. Koller, Peyton G. Russell, Pavi Sreekumar, Bradley R. Teasley, Eva Vandoros, Anastasia M. Zimmerman, Mouna S. DiBenedetto, Christopher A. Korey

**Affiliations:** a Department of Biology, College of Charleston, Charleston, South Carolina, USA; b Department of Biological Sciences, Gettysburg College, Gettysburg, Pennsylvania, USA; Portland State University

## Abstract

Subcluster L3 bacteriophage Finnry was isolated from soil collected in Charleston, South Carolina, using Mycobacterium smegmatis mc^2^155 as a host. The genome of this temperate siphovirus is 75,632 bp long (130 predicted protein-coding genes, 9 tRNAs, and no transfer-messenger RNAs), and BLASTn alignment revealed 99.86% identity with the genome of L3 mycobacteriophage Samty.

## ANNOUNCEMENT

**FIG 1:**
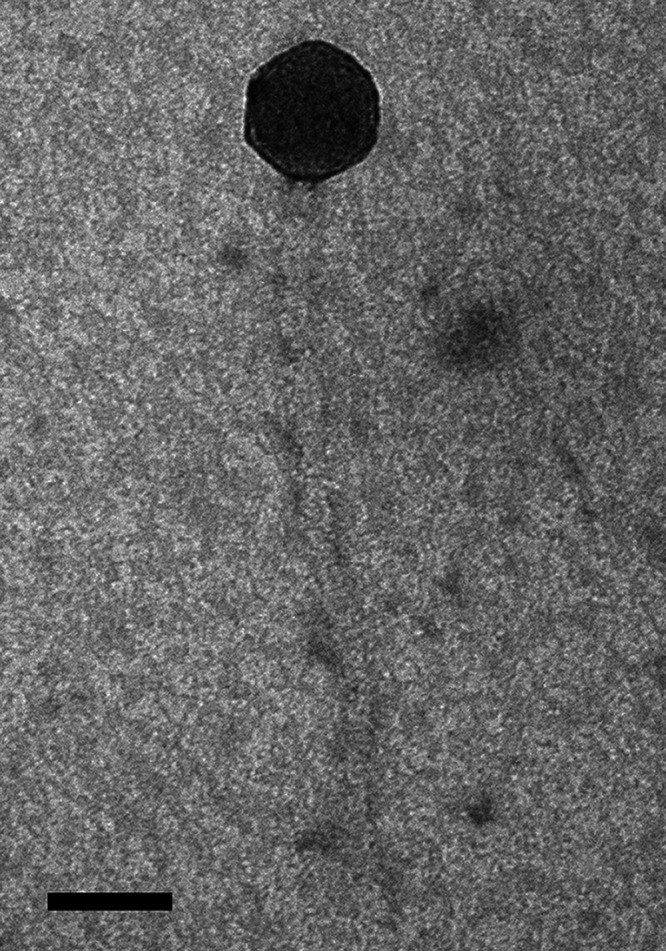
Morphology of the cluster L3 *Siphoviridae* member Finnry examined using a JEOL1010 transmission electron microscope (80 kV). High-titer lysates collected on Formvar-coated copper grids were negatively stained with 1% uranyl acetate (4). Scale bar, 50 nm.

Undergraduates in the Howard Hughes Medical Institute (HHMI) Science Education Alliance-Phage Hunters Advancing Genomics and Evolutionary Science (SEA-PHAGES) program (1) studied the mycobacteriophage Finnry in a broader effort to characterize viral diversity/evolution and improve phage therapy approaches (2, 3). Finnry was obtained from dry, dusty soil at the College of Charleston, South Carolina (32.783445N, 79.937537W), and isolated in Mycobacterium smegmatis mc^2^155 using enrichment at 37°C followed by two purification/amplification cycles in 7H9 top agar, as described in the SEA-PHAGES Discovery Guide (4). Although Finnry forms clear plaques at 37°C, genome analysis indicated that the virus is temperate. Transmission electron microscopy revealed that the phage has *Siphoviridae* morphology, an icosahedral capsid, and a flexible, noncontractile tail (Fig. 1 and Table 1).

**TABLE 1:** Characteristics of the Finnry bacteriophage

Parameter	Data for Finnry
GenBank accession no.	MN096377
SRA accession no.	SRX14989442
Collection site	Charleston, South Carolina, USA
Collection site coordinates	32.783445N, 79.937537W
Isolation host	Mycobacterium smegmatis mc^2^155
Genome size (bp)	75,632
Coverage (×)	1,492
G+C content (%)	59.3
No. of predicted protein-coding genes	130
No. of tRNAs	9
No. of transfer-messenger RNAs	0
Morphotype	*Siphoviridae*
Subcluster	L3
Plaque size (mm) (*n *= 10)	
Range	0.67–1.10
Mean	0.92
Capsid size (nm) (*n *= 3)^*b*^	
Range	54.5–56.8
Mean	55.7
Tail length (nm) (*n *= 3)^*b*^	
Range	286.4–307.7
Mean	299.5
Tail width (nm) (*n *= 3)^*b*^	
Range	13.6–14.3
Mean	13.83
Predicted Finnry protein-coding genes (phams) that are unique to and conserved in all L3 subcluster members (all with no known function)^*a*^	4, 35, 45, 47, 49, 58, 105, 106, 117

a Based on data available in Phamerator on 16 June 2022 (10).

b Measurements acquired from transmission electron micrographs.

To extract genomic DNA from high-titer lysates, the Promega Wizard DNA cleanup system was used, and a DNA library was prepared with the NEBNext Ultra II DNA library prep kit. Pittsburgh Bacteriophage Institute sequenced Finnry on an Illumina MiSeq system (MiSeq reagent kit v3) (5), and 771,310 single-end reads (150 bp) were obtained. Raw reads were assembled into one contig with Newbler v2.9 (6) and verified with Consed v29.0 (7). Finnry’s genome is 75,632 bp, with 1,492× coverage and a G+C content of 59.3%. Genome termini with 3′ single-stranded extensions (5′-TCGATCAGCC) were identified using PAUSE (https://cpt.tamu.edu/computer-resources/pause).

Annotation was performed with the PECAAN (8) workflow tool, and final files were transferred to DNA Master v5.23.2 (https://phagesdb.org/DNAMaster). Programs utilized to identify putative genes included GLIMMER v3.02 (9), Phamerator Actino_prophage v5 (10), GeneMark v3.25 (11), Starterator v1.1 (12), ARAGORN v1.2.38 (13), and tRNAscan-SE v3.0 (14). Functional assignments and domains were detected using BLASTp v2.8.1+ (15), HHpred (16), and the NCBI Conserved Domain Database (CDD) searched with reverse position specific (RPS)-BLAST from NCBI BLAST v2.8.1+ (17) (parameters at https://seaphages.org/forums/topic/5398). Default parameters were used for other software.

Finnry’s genome contains 130 predicted protein-coding genes (51 with assigned putative functions), 9 tRNAs, and no transfer-messenger RNAs. Potential gene duplications include tandem duplication of the WhiB family transcription factor sequences gp79/gp80 (BLASTp indicated 37.66% identity and 79% query coverage) and displaced duplication of gp121/gp131 (BLASTp indicated 42.59% identity and 93% query coverage).

Based on nucleotide similarity, Finnry is classified with similar phages into the L cluster/L3 subcluster, with cluster members sharing >50% nucleotide identity and/or >35% gene content similarity (GCS) (18–20). To compare the distribution of phamilies (phams) (potentially homologous protein-coding sequences sharing >32.5% amino acid identity in CLUSTALW and BLASTp E-values <10^−50^) between Finnry and related actinobacteriophages, Phamerator was used (10). Finnry’s genome contains 9 phams unique to L3 subcluster members and also conserved in all L3 members (Table 1), 2 phams (gp134 and gp137) occurring in only one other L3 member, and 2 phams (gp130 and gp138) unique to Finnry.

GCS scores (19) and whole-genome BLASTn alignments (15) revealed that Finnry’s genome is most similar to that of Samty (93.4% GCS, 99.86% identity, and 99% query coverage), an L3 bacteriophage from Huntsville, Texas. Most L3 subcluster phages (15/16 phages) occur in the southeastern United States (Florida, South Carolina, Louisiana, and Texas) (11 phages) or South Africa (4 phages). Whirlwind is from Pittsburgh, Pennsylvania.

### Data availability.

The GenBank and SRA accession numbers for Finnry are presented in Table 1.
